# Some Points in Laparotomy for Visceral Injuries

**Published:** 1887-07

**Authors:** T. A. M’Graw

**Affiliations:** Detroit


### SOME POINTS IN LAPAROTOMY FOR VISCERAL INJURIES.

BY T. A. M'GRAW, M. D., DETROIT.

[Abstract of a Paper read before the American Medical Association, June 8, 1887.

IT is no longer necessary to urge theoretical reasons in justification of laparotomy for concealed wounds of the abdominal viscera. There are already a sufficient number of successful cases on record to establish the operation as one of recognized merit, and that which now remains to do is to decide upon those technical details, attention to which can alone make the procedure generally successful.

In this paper I shall limit myself strictly to the study of such penetrating wounds of the abdomen as are caused by stabs, punctures and gun shots, omitting all consideration of contused, lacerated and incised wounds. I shall have to deal then, only with such wounds as are distinguished by small external openings, leading into narrow channels, and which are therefore characterized by great and necessary obscurity in diagnosis. Even with these limitations the subject is one of great scope, requiring study from many standpoints. The anatomy of the abdomen and its viscera must be studied with an especial view to operations for the repair of injuries. The very shape of the belly, as it changes in repose and action, may have a decided influence in determining certain practical points. The course of a bullet or weapon through the body and the deviations of missiles from the straight line have to be carefully studied for purposes both of diagnosis and treatment, and the symptoms caused by the various injuries of various organs demand attention. The relations of the external wound and that through the abdominal wall to the internal injuries, the place and character of the incisions to be made by the surgeon through the abdominal wall, the treatment operative or otherwise of the injured viscera, the methods of controlling hæmorrhage and the discrimination of cases which require operations from those in which operations are unjustifiable, are all subjects new as yet to the profession, which give opportunity for much difference of opinion, and which therefore are properly open to discussion. I shall not attempt, however, to do more than to discuss a few of the very many points which would present themselves to the surgeon in relation to this subject.

We may divide the viscera of the abdomen into classes from many different standpoints. They may be divided, first of all, bytheir relations to the peritoneum ; some being completely invested by that membrane while others are either incompletely invested by it or lie altogether without its folds. Wounds of the one kind are marked by extravasations into the peritoneal cavity; those of the other by extravasations into the cellular tissue. We may further divide them into solid and hollow. Solid organs, when injured, may pour into the surrounding spaces their own peculiar secretions and blood from their broken vessels. The hollow viscera may, in addition, pour into the peritoneal cavity or cellular inter-spaces, quantities of foreign matter, which may be undergoing the changes of fermentation or decomposition. Injuries of the solid viscera may cause death by shock, by hæmorrhage, by traumatic inflamations and abscesses, and by suppression of an essential secretion. The injuries of certain of the hollow organs, and especially of the alimentary canal and urinary bladder, may, in addition, cause danger from the septic organisms, which they sometimes contain in great numbers, and which are, in case of injury, let loose upon the defenceless portions of the organism.

While both of these divisions are of certain practical importance, I shall nevertheless discard them in this paper for one which I consider to be of prime importance to the practical surgeon. I shall divide the abdominal viscera according to the degree of mobility which they are permitted to exercise in the abdominal cavity. We find in this respect the greatest differences in the various organs; for, while some—like the ilium and jejunum—have a range of motion which permits them to be drawn to almost any point in the abdominal cavity, others—like the extra peritoneal portions of the duodenum—are bound down to a very limited area. While no one organ can, strictly speaking, be said to be absolutely immovable, nevertheless the motion permitted to certain organs is so small as to be practically of no value. I shall therefore divide the abdominal viscera into the movable and immovable, using these terms relatively and not absolutely, and I shall hope to show in the course of my argument that the two classes require to be so differently considered, when made the objects of operative procedures, as to warrant this mode of classification.

The movable viscera are the spleen, pyloric ends of the stomach and duodenum, the transverse colon, the ilium and jejunum, the sigmoid flexure and the ovaries.

The immovable viscera are the liver and gall bladder, the kidneys and supra renal capsules, the cardial portion of the stomach, the lower three-fourths of the duodenum, the pancreas, the ascending and descending colons and rectum, the uterus and bladder. That even in physiological conditions there are great variations in the mobility of these organs in different people is well known to everybody, while the changes in the length of their ligamentous attachments in conditions of disease are often such as to produce persistent dislocations. Generally speaking, however, and having a special reference to visceral injuries and not to visceral diseases, the lists as given above would be correct.

If we consider the mobility of these organs separately, we may say that the ligaments of the spleen are usually long enough to permit it to be brought over to the median line without great difficulty. The stomach as a whole is quite an intractable organ. It is held firmly in place by the attachments of its cardiac portion, which is always an inaccessible portion of the viscus. The pyloric portion, on the contrary, and the first parts of the duodenum can be moved freely over a pretty large area. The liver, notwithstanding some notable exceptions, is quite solidly fixed in its place under the ribs by short, unyielding ligaments, and the gall-bladder, though more accessible than the bulk of the liver, is scarcely more mobile. The lower portions of the duodenum are not only solidly fixed in their position by the layers of peritoneum which bind them down, but are also among the most hidden and inaccessible of all the abdominal entrails. This gut has no mesentery, and for the most part lies behind the peritoneum. The liver and colons, meso-colons and mesenteric vessels obstruct the operator who is obliged for any reason to get at its interior surface. The pancreas is immovable. The transverse colon is classed among the movable viscera; its range of motion, however, is confined to one direction; its center may often be depressed nearly to the pubis and elevated to the ensiform process, but it can not be moved very far in a lateral direction, on account of the immobile condition of the ascending and descending colons with which it is continuous. When these colons are bound closely to the abdominal wall, the transverse colon has almost no capacity for lateral displacement. When the ascending and descending portions have long meso-colons, the capacity for lateral displacement may be considerable.

The kidneys are of course immovable in their normal conditions. The ascending and descending colons lie in some persons behind the peritoneum and have no mobility whatever. while in others they are partially invested by that membrane, and in others still they have short meso-colons. Even in these last mentioned cases they can be drawn with great difficulty to the median line. The sigmoid flexure and coecum are usually movable with a somewhat limited range of motion, while the rectum and uterus, except in pregnancy or diseased conditions, are usually fixed in place. The urinary bladder may be distended until its upper portion reaches nearly to the navel, but its lower part is quite out of reach from an abdominal wound.

The most movable of all the viscera are the jejunum and ilim, which can be drawn at pleasure to almost any part of the abdominal cavity.

That which makes this one quality of mobility or immobility of such importance to the surgeon is, of course, the necessity under which he labors of making his incisions in the abdominal wall, in case of operations on any viscus, in such a way as to make that viscus accessible. It may be regarded as one of the misfortunes of the surgery of intestinal wounds, that thus far the rules which have been established for the guidance of the surgeon in these cases have been founded on relations which concern only the movable viscera, while those of the immovable have been overlooked or ignored. Of the practical bearing of these anatomical relations I shall presently speak, after a short consideration of the nature of the wounds with which we have to deal.

Stabs and punctures are wounds made by daggers or other long, pointed, thin instruments and are characterized by small orifices and long narrow canals. They may be limited to the abdominal walls or penetrate into the abdominal cavity and injure the abdominal viscera; they vary in depth according to the length of the instrument and the force with which it is impelled. A short weapon may, however, make a very deep wound if the blow is struck with great force. The abdominal walls, yielding to the impulse, are indented by the weapon, and the resulting wound may be altogether disproportionate in depth to the length of the blade which inflicted it. Stab wounds are usually straight, although the blade before its withdrawal may sweep around the circumference of a circle whose center is represented by the external wound ; the narrow orifice may then lead into a wide and destructive wound. While one orifice means that the blade has entered but once, two orifices may either indicate two stabs, or that the one thrust has transfixed the abdomen. If the circumstances are such that the surgeon can know that the two orifices are due to the one thrust, they will then be an invaluable guide to the direction of the wound. The wounds inflicted on the viscera by stabs may be either incised or punctured. Only in rare cases when a dull weapon has been thrust into the abdomen with great force could such a wound be either contused or lacerated.

Gun-shot wounds differ from stabs and punctures in the character of the injury done to the parts and in the greater uncertainty which pertains to the course of the bullet. The size of the missile makes a vast difference in the gravity of the wound, and for this reason the experience gained in war from the destructive effects of rifle bullets on the intestines may not be accepted as an absolute guide in the prognosis when we have to deal with the smaller missiles fired from revolvers and pistols. One wound means of course that the ball is still lodged in the body. The rare exception to this rule is the entrance of a spent ball into the body together with a portion of the clothing and its subsequent withdrawal with the clothing. It might also mean that the surface of the body had been just touched by a glancing ball. Two or more orifices may be made either by one ball passing in and out or by two or more balls, or by one ball, of which broken portions have escaped. When it is certain that only one shot has been fired, two orifices usually indicate the apertures of entrance and that of exit. They do not necessarily indicate, however, that the ball has passed in a straight line from one to the other aperture, for the deflection which it may suffer from the numerous obstacles which it meets may make its course more or less tortuous. These wounds have, indeed, become notorious for their irregularities, and there are many authentic histories of gun shots which have traversed the body in ways so erratic and peculiar as to defy explanation. The study of these cases has produced a faith in their lawless character which is hardly justified by the actual facts. It has indeed so excited the professional imagination as to warp the professional judgment. Putting all fable aside and studying the cases as they have really occurred, we will find that the course of a gun shot is determined by laws as rigid as any to be found in physical science. The deviations which it can make in its passage through the tissues are neither as many nor as great as are popularly supposed, and many so-called deflections are not deflections at all but merely a secondary tortuosity of the wound caused by changes in posture. It may be stated as a general rule, which nobody will probably dispute, that the resistance which can deflect a gun shot from its course must be at least commensurate with the force with which the missile strikes the deflecting surface. It will always be deflected at an acute angle and never at an obtuse or right angle. As regards the size of the angle, the general law will of course prevail that the angle of incidence and angle of reflection will be equal, but this law will be modified in its action by many circumstances, but especially by the peculiarities of the substances upon which the missile strikes. A hard substance like bone may deflect a ball either at a large or at a small angle, or it may partially break and then deflect it, causing complex conditions. A soft and yielding tissue, however, cannot offer sufficient resistance to a gun shot, even though nearly spent, to deflect it, except when it strikes the surface at a very small angle. When the angle is at all large, the momentum of the ball overcomes the resistance and the tissues are penetrated. For this reason balls which run around the body in the abdominal wall always strike it at at a very acute angle, become deflected even when penetrating the skin, and thenceforth until their exit continue to be deflected by the overlying integument at angles so exceeding minute that the whole course of the ball comes to represent a circle of large radius, even then the resistance is finally overcome and the missile escapes. Whether such tissues as form the human intestines, or the softer tissues of the human body, can ever appreciably change the course of a bullet, is with me a matter of great doubt, for the resistance which they can offer is so very small that they must either slip aside or suffer penetration. I am aware that I have the authorities against me in this matter, but the *a priori* considerations against this hypothesis are so strong, and the facts upon which it is founded are so easily explained by other and more reasonable hypotheses, that I feel compelled to regard it as, at least, an unproven theory. It must be conceded that the mere fact that a ball which has pursued a somewhat tortuous course in the body has bruised an intestinal coil without breaking it, does not prove that that intestine deflected the ball. The apparent deflections of the ball may be explained far more easily by the assumption of a subsequent change in the relative positions of the tissues. I think few men, even though medical men, realize how mobile is the abdominal wall and how various are the shapes which it may assume. Accustomed to its rounded form and to the even rythmic motion of breathing, we are apt to forget that every muscle is capable of a separate and individual action, which may altogether alter the shape of the whole. If we observe naked men in violent exercise, such as wrestling, it becomes apparent that the shape of the abdomen in action is ever changing. Now it is flat and again it is round, or one side is flat and the other is round, or one portion is raised and another depressed. The history of phantom tumors indeed, with their deceptive tume factions, is sufficient to show of what the abdomen is capable. Now men are shot often when engaged in violent struggles, and there can be no doubt at all that many a gun shot which has passed through the tissues in an absolutely straight line has been described as deflected on account of subsequent changes in the shape of the body. The only possible way to prove conclusively the power of the human intestines or other similar tissue to change the course of a ball would be to establish the facts by experiments instituted outside of the body, where the influence of all modifying forces can be carefully estimated. It is highly important, in any event, to remember that a soft tissue can not possibly deflect a ball at any but a very acute angle. The human skin is a very tough tissue, but the histories of hundreds of cases in which a ball, striking it at an angle so small that the course of the ball must have been all but parallel to the surface, has nevertheless penetrated it instead of glancing off, will sufficiently show how impossible it would be for that or any softer tissue to escape penetration if the angle of incidence were very appreciable. If this theory is correct the deflections of living balls from the straight line, measured as they must be by very acute angles, can never be very great in the short distance which they have to travel in the body, and in all probability those observations which would seem to prove otherwise should be explained on the hypothesis, not of a glancing ball, but of a subsequent shifting of tissues. Only when the missile strikes a bone can it glance off at an angle of good size. Many of the puzzles which surgeons have encountered are to be explained by the displacements of the dead ball, *i.e.:* of the ball whose momentum has been exhausted. It may then fall to the bottom of the cavity, follow the course of a canal, fall by gravity through the meshes of areolar tissue, or be carried along by muscular contraction. These changes of position do not affect, of course, the nature of the inflicted injury.

A ball entering the abdomen from behind may immediately, after entering the body, strike a bone and glance off at a considerable angle. The aperture of entrance and exit therefore of missile from that direction throw little light on the course of the ball. It is otherwise with those which enter on the front or lateral surfaces of the abdomen. Missiles from in front or the side may deviate, it is true, from their course, and these deviations make it impossible to tell from an examination of the external wounds what organs may have been injured underneath. In this statement I am perfectly agreed with Dr. Parke and other authorities. Unfortunately, however, it has been assumed that because the situation of the aperture and the inital direction of the wounds cannot determine the actual course of the ball through the tissues, therefore the study of these points is of little value to the surgeon as a guide in his operations. I cannot but regard this as a great practical error.

It is true that the exact course of a bullet in the abdomen cannot be determined prior to the operation, but it is not true that its approximate course may not be laid out with sufficient accuracy to guide the surgeon in his external incisions.

The tissues which can deflect a ball entering the abdomen from the front or side are, 1st, those of the abdominal wall, and, 2d, those of the abdominal cavity. When once the ball has traversed the cavity and struck the opposite wall, its power to injure the abdominal viscera usually ceases. There are, of course, exceptions to this rule, where a ball passing obliquely through the abdomen strikes upon a rib and glances to enter again into a viscus, but these exceptional cases are rare. Now the greatest deflection suffered by such a ball before it escapes from the abdominal cavity is that caused by the skin and muscular walls. Should the missile enter these walls at a very acute angle it may run around the whole abdomen and not once enter the cavity. When once it has passed through the abdominal wall, its further course is through soft and unresisting tissues from which it can be deflected, if at all, only at a very acute angle. The course of the ball then from the point where it enters the cavity to that where it makes its exit from it must be nearly in a straight line. Now a careful study of the histories of many gun-shot wounds of the abdomen in which the ball has entered from the front or at the side has convinced me that the course of a bullet through the cavity and its wall lies almost always in the same plane, so that a plane projected to any three points in its course will have within its limits the whole path of the missile. I do not mean to be understood, in saying this, that the path and the plane would coincide with a mathematical exactness, but that their coincidence would be near enough for all practical purposes.

The especial purpose of these studies is to determine, if possible, how the surgeon can best make his incision through the abdominal wall so as to arrive easily and quickly at the seat of visceral injury. The best men in this department of surgery have already agreed where the incision should be made and where it should not be made. Parke, Dennis, Bull, Morton and Nancrede all lay down the law to the effect that the incision must be made in the linea alba, and that the wound must not be enlarged nor disturbed except for purposes of exploration. I have as yet sought in vain for any adequate explanation of this rule, although the unanimity with which it has been adopted is something marvelous when we consider the independence which surgeons manifest in other departments of surgery.

Parke states positively "that primary abdominal section in the mid-line gives the best command over the damage done and furnishes the most feasible opening through which the proper surgical treatment of such damage can be instituted." "The wounds of entrance and exit of the bullet *should not be disturbed* in any manner except to control bleeding or remove foreign bodies when present. They need only to be covered by the general anti-septic dressing applied to the abdomen." And Dennis says: "The incision should be made in the median line. Any deviation from this rule is objectionable in many respects. The side incision does not permit the free exploration of the cavity, involves also dividing planes of muscles, the hæmorrhage form, which is troublesome to control, and the primary union difficult to secure. The median incision divides no vessels of any importance, passes through the linea alba, which can be securely sutured, and is not likely to give rise to ventral hernia." "The enlargement of the original wound for an examination of the peritoneal cavity will not enable the surgeon to exclude in all cases faecal extravasation, perforation, volvulus or hæmorrhage." And Dr. Nancrede solemnly warns the practitioner never to enlarge the original wound unless it is located in the linea alba.

I confess that I have been unable to recognize in these arguments that decisive logic which solves all doubts, and I find myself in that most depressing of all situations, in a minority of one. *Why* should the wounds of entrance and exit never be disturbed? *Why* should the incision never take in the original wound unless that chance to be in the median line? *Why* may not the surgeon explore the abdomen with sufficient thoroughness by a side incision, if he make it long enough? There is no point in the abdomen through which an incision cannot be made as long as the linea alba in a straight line, if the surgeon chooses. Muscular hæmorrhage may be troublesome, but it is not usually difficult to stop, and incisions through muscular tissues heal by first intention, as those gentlemen can testify who have had experience in operations on the stomach, in which the incisions have been made parallel to the ribs through muscular planes. I have yet to learn that those operations have been followed by an undue number of ventral hernia. But while the reasons adduced for forbidding all but the median incisions are not convincing, there are many very cogent reasons for departing from that rule in many cases. Take for instance the case of any one of the immovable viscera. It may not, in case of injury, be possible to reach such a viscus from the median line, except by an enormous incision, and it may be possible to reach it by a short cut if made in its neighborhood. Are we prepared to accept the doctrine that a cut of twelve inches in the linea alba is less dangerous than one of four in the muscular wall ? I had myself, last winter, a case of gun shot of the ascending colon and coecum which will illustrate this point.

Maggie McMahon, aged 24, was shot in the abodmen, at three o'clock on Tuesday morning, Dec. 21st, 1886. She was carried to St. Mary's Hospital at half past eight o'clock, and I saw her at nine o'clock on the same morning. I found her vomiting bile, and complaining of great pain. Her pulse was 120 per minute, her respiration 30 and her temperature 120° F. The bowels were moderately bloated and very tender, and two inches above and two inches inside of the right anterior superior spine of the ilium there was a gun-shot wound with black edges. There was no tympanitic resonance over the liver, no extravasation of faecal matter, no emphysema, and no general meteorism. The pain was located in the hypogastric right iliac and right lumbar regions. The wound was supposed to have been made by a ball from a revolver of 32 caliber, although that point was never fairly settled. At 12 o'clock, noon, I cut through the abdominal wall at the site of the wound, by making an incision of four inches long in the direction of the fibres of the external oblique muscle, the wound being in the center of the incision. It was easy to trace the wound into the abdominal cavity by the discoloration of tissues, and it soon became evident that the ball had passed from before backwards, and from above downwards, into the peritoneal sac. My finger, on entering the cavity, immediately detected a hole on the anterior surface of the ascending colon, which I thereupon drew out on to the abdomen. This wound, from which faecal matter was constantly pouring, was an oval with its long axis transverse to that of the intestine. It was about half an inch long and a quarter of an inch wide. I sewed it up with a continuous suture of cat gut, in a double row, after Czerney's modification of Lembert's method. Searching then for the wound of exit, I found it also vomiting faeces directly behind at the junction of the meso-colon with the intestine. The meso-colon and meso-coecum in this case were long as the peritoneum, completely surrounding the gut. The ilium was united to the intestine on its inner and posterior surface midway between the two wounds. The second wound had its long axis also transverse to that of the bowel, and was sewed together in the same way as the first. This done, I was able to trace the track of the bullet down into the femoral canal before the external iliac and femoral artery, by a ragged opening in the muscle, but its course afterwards is a matter of great uncertainty. I carefully examined the small intestines without withdrawing them from the cavity, but found no other wound. The abdominal cavity was washed out and closed with wire sutures, and a drainage tube inserted The patient began immediately to improve, and her temperature fell a degree on the same day ; she vomited less frequently, and the ejecta contained only mucus and swallowed fluid. Her pulse fell to 100, but her temperature rose again on the second and third days to 102. After the third day the improvement was more marked, and after sixth day she ceased altogether to vomit, and her temperature fell to 99 and 100. The drainage tube was then withdrawn, without having discharged one drop of pus. Subsequently, however, a small superficial abscess formed in the track of the wound, and on the tenth day discharged a teaspoonful of pus. Her convalescence was thenceforth uninterrupted, and she left the hospital Feb. 2d suffering only from a lameness consequent upon the passage of the ball into the thigh. There were present at the operation, Dr. Perley, U. S. A., Dr. H. O. Walker, Dr. N. W. Webber, Dr. Shurley, Dr. Boise, Dr. Robbins and others.

Now in this case I called the attention of the gentlemen present to the difficulty which I should have experienced in reaching the wounds in the gut, and especially that one on its posterior surface, had I made the usual incision in the linea alba. It would have been simply impossible for me to have got at it without cutting from the ensiform cartilage to the pubes and eviscerating her, whereas with a short incision I was enabled with ease to perform the necessary manipulations on the bowel and to carefully examine all that was necessary of the small intestine. I have since several times on occasion of post mortem examinations endeavored to bring the ascending colon out through an incision in the linea alba as it would have to be drawn out for purposes of operation, and have every time found it to require a long incision, and even then to be a matter of great difficulty. Here, then, was one case, at least, in which a great error would have been committed by adhering to the arbitrary rule of median incision. But that which was found true of the ascending colon would be found to apply equally to the descending colon, stomach and liver. There is no way in which an immovable viscus can be made so accessible as by an incision parallel to its long diameter.

Even in operations upon the pylorus, which is quite free and movable, many surgeons have preferred the transverse or oblique incisions to that in the linea alba, because, as Wolfler says, 1st., The diseased portion is more readily got at ; 2d, the remaining portions of the stomach can be more easily examined; and 3d, the existing adhesions to the peritoneum can be more easily excised. And besides these, for the further reason that the intestines are less readily prolapsed than in the median incision (Dr. E. Kuh, Langenbeck's Archives, 27th Vol., 24th part.)

How much more, then, than in excisions of the movable pylorus, is the transverse or oblique incision indicated, in those gun-shot wounds of the stomach in which both walls are perforated, and in which the posterior wound may lie very near the cardiac orifice. In such cases, the incision perpendicular to the long axis of the organ puts the surgeon to a great disadvantage, while a cut transverse or oblique or even slightly circular under the ribs enables him to expose the whole organ and draw it out into view. The perpendicular cut in the linea alba in case of gun-shot wounds of the liver enables the surgeon to feel the liver's edge and to pass his fingers for a short distance over its surfaces. He can neither examine the organ as a whole nor inspect its lower surface, while a transverse cut just below its edge will bring the whole viscus into the field of operation and enable him to operate even on its under surface. Let us apply these principles to gun-shot wounds. The first points to determine are whether the wound is a penetrating wound, and if so, what viscera have been hurt ? Without discussing the question of diagnosis, on which I have nothing new to offer, I will say that it is generally conceded that there is no other way to determine the question of penetration except by following the track of the ball until it either enters the abdominal cavity or leaves the abdominal wall; or if the track is too long, then by boldly opening the abdomen elsewhere for purposes of exploration. The question of whether to follow the one or the other of these procedures must be left to the judgment of the surgeon. It is evident that one could not follow the track of the ball if it lead across the whole width of the abdomen. It is equally clear that one ought not without due cause, to open the peritoneal cavity. Probably the best rule to follow is for the surgeon, in cases of doubt, always to begin his work by exploring for a short distance the track of the ball. If, then, it leads him quickly into the abdominal cavity, he learns that fact and can proceed accordingly. In any event, he learns the initial direction of the missile and can form some judgment as regards its further course. I think that the profession have undervalued the practical worth of the knowledge thus acquired. The conviction has been so strong that the ways pursued by gun-shots were utterly erratic and unaccountable that surgeons have come to discard all indications as of no service in actual diagnosis. This extreme view is certainly not justified by any larger experience. That no man can say before exploration exactly what organs are wounded is certain, but any surgeon with good judgment, after a careful examination of the course of the ball through the abdominal wall, can say about where the missile is gone and what viscera it may have hit. The course of a ball from the time it enters the abdomen until it strikes the opposite wall *must* be nearly in a straight line, for the reason which I have mentioned, and if it were not for the shifting of tissues and changes in shape which occur subsequent to the shooting, comparatively little difficulty would be experienced in tracking the bullet. As it is, the initial course of the ball reveals much that is of inestimable advantage to the operator. If a bullet enters two inches to the right of the navel and passes from left to right and downwards and backwards, the surgeon knows positively that it has not hit the spleen, nor the stomach, nor the liver, if these organs are of normal size and in normal position. He knows absolutely, too, that such a ball could not possibly hit any organ which did not, at the time of the shooting, occupy a position downward, backwards and to the right of the aperture of entrance. In this hypothetical case, it might hit the transverse colon, or the small intestine, or the ascending colon, or coecum, or a mesentery, or meso-colon, or some blood vessel. Now an incision through the abdominal wall, beginning at the wound and carried a sufficient distance downwards and outwards in the line of its track, would suffice to enable the surgeon to examine every organ injured and to repair every injury. Every injured viscus could be brought into the wound and no viscus which could not be brought into the line of the incision could possibly have been injured by the missile.

The rule, which seems to have met the general approbation, that every inch of small intestine must always be examined in a gun shot wound of the abdominal cavity must surely be reconsidered. How, for instance, in such a wound as I have described, could the duodenum possibly be injured at its pyloric end? How, in a gun shot above the navel, in which the course of the wound passed from before and below, backward and upward, could the missile possibly come in contact with the lower part of the ilium? How, in any wound, could any viscus have suffered injury, which could not by gentle traction be brought into view through an incision, which, beginning at the aperture of entrance, uncovered the whole course of the ball? Let us take another hypothetical case, that of a gun-shot wound of the right hypochondriac region, in which the ball entering three inches above and two inches to the right of the umbilicus, passes obliquely upward and backward. In this case, while it may not be beyond the bounds of possibility that a coil of the jejunum or ilium might be wounded, yet such an event would be extremely improbable, would it then be good practice for the surgeon to haul out these viscera into the cold in order to satisfy himself that a wound *did* not, which in the nature of things *could* not, exist ?

Two cases from the current literature will illustrate this better than any hypothetical cases. In one case, a ball entered a little to the left and above the navel. With the accustomed indifference of surgeons nowadays to the course of the wound, the wound does not seem to have been examined in order to find out its direction. From the fact that the ball perforated both walls of the stomach and both walls of the duodenum, it is fair to infer that it passed from below and before upwards and backwards, and from left to right. Notwithstanding that it was utterly impossible for such a ball to injure any of the lower parts of the intestines, the whole intestinal track was examined down almost to the ileocæcal valve.

In case second, the ball entered over the ninth rib three and three-fourths inches to the right of the median line and four and a half inches above the navel (there must surely have been some mistake in these measurements), and was found one and one-half inches above the navel and eight inches to the left of the median line. It grazed the liver, perforated the stomach, wounded the small intestine in the left flank (the jejunum) and lacerated the left kidney. The surgeon subjected the entire bowel from the stomach to the sigmoid flexure to a close examination, but why? The lower portions of the duodenum, the greater part of the ilium, the ascending colon and cæcum and the lower part of the descending colon and sigmoid flexure could not possibly have lain in the course of the ball. Both of these cases died, and it may not be impertinent to ask if the unnecessarily prolonged manipulation and exposure of the intestines had anything to do in causing their deaths? I do not wish, in criticising these cases, to be understood as reflecting in any way upon the practice of the two surgeons, both of whom I greatly esteem. They were perfectly justified in their manner of operation by the prevailing professional public opinion. I wish, however, to express emphatically by opinion as to the utter senselessness of a rule which requires the surgeon to examine and expose viscera and portions of viscera which could not possibly, in the nature of things, have suffered injury. The only explanation, indeed, which I can make for its adoption is the queer belief, apparently prevalent in the profession, that a gun shot has the power to run around the abdominal cavity, regardless of all natural law.

It is curious how the adoption of a theory will influence men's ideas and practice. I believe it is a well established rule in abdominal surgery that the longer the incision the greater the danger. Lawson Tait's great success had been ascribed by some to his remarkable skill in operating through small incisions, and yet, in the matter of operating for intestinal wounds, surgeons have seemed to persuade themselves that it made no manner of difference whether the abdomen was split in the median line from ensiform cartilage to pubes. Dr. Morton says, indeed, that there *is* no harm in a long wound if properly treated. I must protest earnestly against this kind of teaching. There is harm in a long wound even though properly treated, and when the surgeon makes one longer than is necessary he thereby unnecessarily endangers the patient's life. It is not only that the danger of infection is greater in a long wound, but also the danger of shock, the danger of chill, the danger of prolonged ætherization, and the danger of exhaustion. I have had many a patient under my hands whose life or death seemed to me to depend upon the time in which the operation could be got through with. I do not mean to say that the surgeon should ever allow himself to make an incision too short to accomplish his purpose. On the contrary, I strongly suspect that hitherto, while the incisions in the linea alba have been in some cases made too long for safety, those which have been made in the track of the wound have been too short to be effectual.

I have tried by studying the cases already published to come to some definite conclusion as regards the relations which are borne by the directions of the wounds in the abdominal walls to the wounds of the viscera underneath, but have failed to come to satisfactory conclusions on account of the defective character of the reports. In most of the reports the position of the wound is given, but its direction through the abdominal wall is altogether ignored—a circumstance which indicates forcibly the neglect into which this most valuable indication has fallen. Before we can, however, come to a conclusion as to what wounds demand median incisions, and what wounds demand incisions elsewhere, what wounds demand examination of the whole intestinal track, and in what wounds that tedious and dangerous examination can be dispensed with, we must have at our disposal large numbers of accurate records in which the position of the external wound, its course through the abdominal wall, its subsequent course through the cavity of the abdomen and its termination, together with all other details, are reported so exactly as to permit of critical study. In the meantime, every surgeon who operates must be left unhampered by arbitrary rules, to make incisions and to follow operative methods according as his judgment dictates in any individual case.

When the abdomen is opened the visceral wounds discovered must be repaired. In considering these repairs, we have to distinguish between those of the hollow and those of the solid viscera. Injuries of the intestinal wall must be invariably sutured with the Lembert suture. Many efforts have been made to improve this suture without success, unless Czerney's modification may be so considered. I have myself made very many experiments in this direction, which I will note here, for the purpose of pointing out a false way, into which some one else might blunder. I conceived the idea, after reading accounts of cases in which the Lembert suture had failed to hold the wounds together, that the intestine might grow together more rapidly and with a stronger union if the opposing surfaces were made raw by denuding them of their serous coverings. On the thick intestinal wall of the dog this is quite practicable. The serous membrane can be easily dissected with the finger nail from the muscular wall, and there will be left a thickness sufficient to hold a fine suture without having the needle pass through the mucous membrane. I operated on a great number of dogs after this fashion. Opening the abdominal wall, a gut was seized and brought to the surface. A circular cut was then made through the serous coat of the gut. This was dissected up on each side of the cut, and turned back like the cuff of a coat. It was wonderful to note the contractile power of the serous coat when freed from its attachments. It would then contract to half its previous dimensions. When the serous coat had been turned back, the intestine was cut through and the remaining walls cut partly away. The resected ends were brought together by sutures passed through the muscular layer alone, and then a second row through the shrunken and sometimes curled-up serous coat made the union secure. In some cases I, at the same time on the same dog, made two resections, one by this method and the other by Lembert's method, in order to compare the two kinds of stitches. The most of my dogs survived the operation, and as far as that goes my operation could be considered a success, but a careful comparison between my own method and Lembert's proved conclusively the superiority of the latter. The serous surfaces not only healed together with less inflammation and suppuration than the raw, but the union could, after the same lapse of time, endure a greater strain.

Besides that, I satisfied myself on the cadaver that my method was quite impracticable on the thin-walled human intestine, and I abandoned it. I do not believe that any other method will ever supplant that of Lembert, and the only question is, which of the various modifications is most useful. I have adopted a continuous suture which I believe will make the patient nearlyabsolutely secure against any failure in union or extravasation of fæces. In the case I have just reported, I began the suture at a little distance from the end of the wound, by carrying the thread through the serous and muscular coats and then knotting it, leaving the end long. A continuous suture was then carried along the whole course of the wound and a third of an inch beyond it. The suture was then carried back at a higher level after Czerney's method until the point of beginning was reached, and then the thread was drawn tight and the two ends tied firmly together. It does not matter what kind of a continuous suture is used if the insertions of the thread are near enough together. The continuous suture is in my judgment better than the interrupted, both on account of its greater security and because it can be applied much more quickly.

There is another question in connection with these intestinal wounds, and that is, what wounds, if any, require resection of the bowel? Madelling had shown conclusively that any considerable destruction of the mesentery will insure gangrene of that portion of the bowel whose afferent blood vessels are thus destroyed. The case of Dr. Keen, reported at the last meeting of the American Surgical Association, corroborates Madeling's observations. If I understand his description, he found on post mortem examination, opposite the injury to the mesentery, a gangrenous spot as large as a five-cent piece.

Pending further experience on this point, we may say then that great injury of the adjacent mesentery may demand excision of that portion of the bowel with which it is in contact. I do not believe that any other form of injury can ever demand excision, or be treated by any other method as safely and successfully as by suturing the wounds. Even such multiple wounds as Parke describes, and for which he counsels excision, will heal perfectly well by simple suture. Some experiments made upon dogs by myself and my assistant, Dr. F. W. Robbins, will illustrate this point. My object in operating was to find out what could be done by suturing multiple wounds near to one another in a gut instead of resecting the gut. I therefore in each case drew out the small intestine and cut holes with a pair of scissors from one to two centimeters in diameter. In one case they were made six in number, on one and the same side of the gut. This animal recovered and was afterwards operated upon by having two holes cut, each a centimeter in diameter on opposite sides of the gut and directly opposed. He recovered a second time.

In a second animal four holes were cut near one another but irregularly placed. This animal got well.

A third, operated on on a dark day, died, and the sutures were found to have been imperfect. There were in all six operations followed by one death. In closing the intestine, it was drawn together in such a way that the edge of the wound was transverse to the long diameter of the gut. In this way, the lumen of the gut was enlarged instead of narrowed at the seat of injury, whereas if it had been made parallel to the longitudinal axis, the lumen of the intestine would have been lessened. If a wound were so located, however, that its edges could be brought together only in a longitudinal line, the surgeon could nevertheless close it in this way without fear of obstruction. Mikulicz closed in one case the defect in the bowel by a longitudinal suture, which made a considerable narrowing of the gut at that point. The patient nevertheless recovered, and suffered no subsequent symptoms of obstruction. Few people seem to realize the elasticity of the intestinal wall. It will stretch, especially in a longitudinal direction, like india rubber, and this elasticity enables it to recover its tone and shape after injury and loss of substance. For this reason I must dissent from the practice which has been recommended of excising the intestine for multiple wounds. Where the interval between the two wounds is too small to permit the insertion of stitches, the bridge should be cut away and the two wounds turned into one. But, however many the wounds, they should all be sutured without resection and the operation of resection reserved for those cases in which the vascular connections of the gut have been widely destroyed.

Wounds of the solid viscera have not yet been subjected to sufficient experimental study to enable the surgeon to form an opinion as to the best method of treatment. I have, myself, operated on three dogs by incising the edge of the liver and closing the gap by deep sutures. The bleeding was not very considerable. I passed sutures of fine silk or cat gut deep into the liver substance and thus closed the wounds. On one of the animals, an operation was simultaneously performed on the stomach. This one died after 24 hours. The edges of the cut liver had become agglutinated. The other two recovered.

The slight consequences, comparatively speaking, which have followed incisions into the liver for abscess should give rise to good hope in case of a wound of this organ not involving the large veins. The treatment of such a wound should be either by suturing it finely and deeply with cat gut, or by stuffing the wound in the organ with iodoform gauze, and keeping the wound in the abdominal wall open for drainage. This last course was pursued successfully by Dr. H. Burckhardt, of Stuttgart. This surgeon was called on March 29th, 1886, to see a man who was collapsed from a stab it the mamillary line just below the ribs. As the patient was evidently dying of internal hæmorrhage, Burckhardt made a cut in the abdominal wall of 12 to 14 cent. in length, beginning at the wound; found the abdomen full of blood, the source of which proved to be a wound three cent. long in the left lobe of the liver. He stuffed iodoform gauze into the wound and stopped the hæmorrhage by compression. The patient recovered.

Whether a gun-shot wound of the kidney or spleen should demand excision of the organ is a question which further experience must answer. Wounds of the pancreas should be treated according to the principles laid down by Dr. Senn in his admirable monograph on that subject.

I shall in this paper discuss only one more question, that, namely, as to the class of penetrating wounds on which the surgeon should refuse to operate. The rule to perform laparotomy for penetrating wounds will surely be found to have many exceptions.

One of these exceptions may or may not be the wound of an empty stomach. A case from my own practice will illustrate this point.

Thomas J. Hilbish, a medical student, was the happy possessor of a revolver, which he was accustomed, at night, to keep under his pillow. He arose on the morning of July 17, 1879, and, for some reason, jerked the sheet from off the bed. With the sheet came the revolver, which fell upon the floor, and discharging one of its barrels. The ball struck Hilbish a little above and to the left of the navel, and its course was from below, upwards and, presumely, to the left. I was called and arrived within half an hour. He had then vomited blood and once again he vomited blood. He had eaten a light supper at six o'clock and had taken nothing into his stomach since. The wound was not probed, but from the position in which he stood at the time of the accident, and which was described on the spot, I judged that its course through the tissues was from right to left, from below upwards, and from before backwards. He was put to bed, placed under opiates, allowed for several days neither to eat nor to drink, was fed and watered by enemata, and he recovered, not, however, without suffering from a severe traumatic peritonitis, during which he experienced great pain and his bowels became enormously bloated. In this case, there can be little question that the stomach was wounded, and that the time of injury in the early morning, when the viscus was empty, had everything to do with the subsequent favorable course of the wound. Dr. Keen's case illustrates probably (for the condition of the stomach, as respects its contents, is not noted) the same point. The girl shot herself at 6:30 A. M., and we may presume that she was then fasting. Dr. Keen especially remarks that there was no extravasation of the contents of the stomach and the wounds could hardly be discovered. The stomach is indeed much more tolerant of injury than is usually supposed. A remarkable case was that of Professor Von Mosetig, published in the *Vienna Medical Wochenschrift* of Dec. 18, 1886. On Nov. 17, 1886, a man of thirty-one years shot himself in the left side, the ball entering the fourth intercostal space a little outside of the nipple. There was no wound of exit, but in the back between the tenth and twelfth ribs there was a hard spot. The patient showed no gastric distress, no tenderness of the bowels and no vomiting. He had hæmatothorax on the left side and suffered greatly from dyspnœa. During four days he had no fever and ate heartily and regardlessly. On Nov. 21st he was seized with erysipelas of the face, from which he had on Nov. 30th recovered. He remained free from fever for the next two days, when on the third day a rise of temperature preceded the discharge through the wound of a large quantity of offensive pus and blood. The hard spot on the back was then opened and the bullet found and extracted. The finger extended here into a cavity containing a large amount of black putrid matter. No communication could be established between the two orifices, but when the patient vomited on awakening from the anæsthesia, he discharged from the stomach some of the permanganate of potash solution which had been injected into the wound. After that food was from time to time discharged through the posterior wound. The patient remained free from fever, but on December 8th died suddenly from causes not stated in the narrative. The adduction revealed a communication of the anterior opening through the cavum pleuræ, diaphragm and left lobe of liver, with the stomach, both of whose walls had been perforated. Here, then, had been a frightful wound of the pleural cavity, stomach and liver, in which the only symptoms of trouble, during several days, had been those caused by hæmorrhage into the pleural cavity. Von Mosetig assumed that the stomach, at the time of injury, had been empty, and had quickly formed adhesions to the surrounding organs.

I have myself, in connection with Dr. Robbins, made a few experiments on dogs in order to determine the effect of wounds on an empty stomach. The dogs were all kept fasting for twenty-four hours, and then operated on by drawing the stomach out, stabbing it in one or more places and then returning it unsutured into its place. The wound in the abdominal wall was then securely sutured and the animal kept fasting for 48 hours with sparing allowance of water. Feb. 25, 1887.

Case 1st: Moderate sized dog, fasting for 24 hours. The stomach was drawn out and both anterior and posterior walls perforated simultaneously by passing a scalpel through them. This double puncture was made twice, with an interval of half an inch. The stomach was then replaced and the abdominal wound sutured. The animal was then kept fasting, but was allowed a little water. After 48 hours he was fed on milk. He recovered without a bad symptom. The wounds through the stomach walls were about half an inch long.

Case 2d: Black dog, moderate size, fasting, was operated on as above, making, however, but one double wound. Died 24 hours after operation. There were found on post mortem evidences of great paritoneal congestion. In the abdominal cavity there was about an ounce of bloody fluid. The stomach was half filled with thin, yellowish fluid. The anterior wound had healed except that one corner, where there was an opening as large as a pin's head. The opening in the posterior wall, where the knife had been passed, had so thoroughly healed that even its location could not be discovered.

Case 3d. Feb. 26, 1887:

Dog of medium size, fasting, was operated on as before by grasping the stomach and transfixing both walls. This dog made a quick and complete recovery. The hole in the stomach was one cent. long.

Case 4th. Feb. 26, 1887:

Dog operated on as above, but the incision in both anterior and posterior walls measured half an inch. This dog recovered.

Case 5th. Scotch terrier. I operated on this dog as above, and besides cut a gash an inch long in the edge of the liver. He died in 26 hours. About three ounces of red fluid was found in the peritoneal cavity. The wound in the liver had become agglutinated outside, but not in the depths of the wound. The wound in the anterior wall of the stomach could not be detected from its peritoneal aspect, but the mucous membrane was marked by a linear slit. On the posterior wall, a discolored spot on the peritoneam showed the site of the wound, which, however, was closed, but the mucous surface had not healed.

It was astonishing to note, in the cases of death, how quickly nature had repaired most formidable injuries, for in both cases one of the wounds had healed so thoroughly by first intention that its site could be with great difficulty detected.

It can not be denied in the face of such evidence that wounds of the empty stomach, through perforating both anterior and posterior walls, may have a good prognosis, and yet I should hardly venture to say that the penetrating wounds, which are located in the region of the stomach and involve that organ, should be excepted from the general rule of exploratory laparotomy.

The difficulty of excluding injury of other viscera, and especially of the liver and colon, would seem to make abdominal sections imperative even in those cases.

When, however, from any other cause, the operative procedure should seem to be of doubtful propriety, the facts which I have just related might make the expectant treatment more jusifiable.

A condition which would seem to me to more positively forbid abdominal section is that of great obesity. The case reported by Dr. Britton illustrates one objection to operating on such cases, namely, the difficulty in performing the operation through several inches of fat. The surgeon in such a case is cramped for room in which to work, and is further hindered by the difficulty in distinguishing the various tissues within the abdominal cavity. This is so great in cases of vast intra-abdominal accumulations of fat that physicians are puzzled even in post mortems to distinguish the relations of organs. How much more so must be the case in operations during life in which the external incision must be limited!

There is, however, an objection, even more weighty, to these operations, and that is the difficulty of securing an absolute asepsis where the necessity of the case forces the surgeon to break an innumerable number of fat cells and to leave n the abdominal cavity an immense quantity of disorganized free fat. With all that, he has to do with a patient who belongs to a class notorious for their slight powers of resistance to traumatism. For these reasons the outlook of operations, especially on persons in whom there are likely to be multiple wounds of the small intestines, is gloomy enough. I do not mean to say that such cases are to be always considered as inoperable, but that the accumulation of fat must be reckoned among the circumstances unfavorable to operative procedure.

The persistence of great shock, if only it could be distinguished from the collapse caused by internal hæmorrhage, should contra-indicate the operation for the time, but as the differential diagnosis between the two conditions is often impossible, the surgeon will frequently find himself in a dilemma, between the indications caused by a possible hæmorrhage or those caused by a possible condition of shock.

The occurrence of peritonitis, although of disastrous import, should not in itself, in my opinion, contra-indicate laparotomy.

In conclusion I wish to urge upon the profession the consideration of the following propositions, not as dogmas for adoption, but as theories and facts for discussion:

1st. There are many viscera in the abdomen which are practically immovable —so immovable as to forbid operations upon them through the median line. When a gun-shot wound is so located and so directed as to make the injury of an immovable viscus probable, the external incision should be made with reference to that fact.

2d. The course of a gun-shot wound is determined not by chance, but by the operation of immutable laws.

3d. When a gun shot is deflected from its course, it is always at an acute angle, and, when deflected by a very soft substance of little resistant power, the angle of deflection must be exceedingly small. When, therefore, a gun shot passes into the abdominal cavity, the deflection of the ball from the time it leaves the aperture of entrance until it strikes the opposite wall, can not be sufficient to appreciably alter its course.

4th. The initial direction of the ball through the abdominal wall indicates very nearly its subsequent direction through the abdominal cavity. The careful study of the wound of entrance is, therefore, of the greatest importance, and no surgeon should open the abdomen for the repair of visceral wounds without first exploring the wound of entrance sufficiently to make sure of its course through the parieties.

5th. The ball, after passing through the abdominal cavity, may be deflected by the bones or soft tissues; but as it, in the vast majority of cases, pursues the remainder of its course outside of the abdominal cavity, these deflections should have no influence in determining the line of incision.

6th. Gun-shot wounds of tortuous course frequently owe their apparent deviations from the straight line to a change in the shape of the abdominal wall subsequent to the shooting.

7th. In general, the course of the bullet through the abdominal wall, prolonged by a line drawn on the external abdominal surface, will indicate the course of the bullet through the cavity if its velocity is great. If at angles of five or of not more than ten degrees with this line, two other lines are drawn on either side of it, beginning at the wound of entrance, we will have represented on the abdominal surface the greatest deflections of which a bullet is capable during its passage through the abdominal cavity. An incision, therefore, along the whole length of the first straight line could not fail to uncover every part of the course of the ball, provided that there has been no subsequent displacement of the injured tissues.

8th. For this reason an incision, beginning at the wound of entrance and prolonged to a sufficient distance in its course, is often the very best which the surgeon can make. This is especially true of such wounds as, beginning at a distance from the linea alba, pass in a direct line away from that line.

9th. The immovable viscera can be best exposed by incisions made parallel to their long diameters.

10th. When abdominal section is made for penetrating wounds, every organ and part of an organ which could possibly have lain in the path of the ball or weapon should be thoroughly explored by the surgeon, but to examine an exposed viscera or portions of viscera which could not possibly have been injured would be an error in practice.

11th. The prognosis of wounds of the empty stomach is not necessarily bad.

12th. Large accumulations of fat in the abdominal wall and cavity may sometimes contra-indicate laparotomy for visceral wounds.

